# Causes of Kawasaki Disease—From Past to Present

**DOI:** 10.3389/fped.2019.00018

**Published:** 2019-02-05

**Authors:** Satoru Nagata

**Affiliations:** Departments of Pediatrics, Tokyo Women's Medical University, Tokyo, Japan

**Keywords:** Kawasaki disease, etiology, superantigens, heat-shock proteins, epidemicity

## Abstract

Kawasaki disease (KD) is a multisystem vasculitis that primarily affects the coronary arteries of young children. The causes of KD remain a mystery. It is suspected that some sort of infectious agent is involved because KD has epidemicity and seasonality. That said, the incidence of the disease is high among Japanese people, so it can be speculated that the hosts may have some sort of genetic characteristic that leaves them susceptible to KD. Various theories regarding the etiology have been asserted, such as the infectious vasculitis theory, autoantigen theory, superantigen theory, and RNA virus theory; however, none of them have been able to overcome this epidemicity. Taking into consideration the knowledge gained from previous reports, the best scenario explaining the pathogenesis is “individuals with certain genetic backgrounds are affected by microorganisms which trigger KD.” In this article, the pathogenesis of KD is discussed with a focus on the microorganisms mentioned above, along with the previous and current hypotheses as well as my own opinion.

## Introduction

The etiology of Kawasaki disease (KD) has remained a mystery since Dr. Tomisaku Kawasaki proposed the disease in 1967. A number of epidemiological and clinical observations suggest that KD is caused by an infectious agent, with suggestions ranging from Staphylococci, Streptococci, Mycoplasma, or Chlamydia (1–4), to viruses such as adenovirus, parvovirus, or Epstein-Barr virus (5–7). It is suspected that infection is involved because KD has epidemicity and seasonality. That said, the incidence of the disease is high among Japanese people, so it can be speculated that hosts may have some sort of genetic characteristic that leaves them susceptible to KD. Taking into consideration the knowledge gained from previous reports, the best scenario explaining the pathogenesis is “individuals with certain genetic backgrounds are affected by certain microorganisms which trigger KD. This article provides an overview along with the newly acquired knowledge by Nagata et al. (8), with a focus on +pathogenic organisms.

### Possible Pathogens

Various pathogens have been proposed as the trigger, but none have been decisively established. One of the reasons is that there are a variety of items that must be explained to determine that the etiology is certain [Table 1, (8)]. In particular, the epidemiology, wherein “the incidence is high among Japanese people,” shall be most difficult to explain.

**Table 1:** Epidemiological conditions that the pathogen of Kawasaki disease must meet (8).

**1. EPIDEMIOLOGICAL CONDITIONS**
Frequently observed in infants aged four or younger
Moderate epidemicity
Frequently observed in Japanese people
**2. PATHOLOGICAL CONDITIONS**
Includes six types of characteristic clinical symptoms
Redness in the BCG region
Cures within 2–3 weeks (self-limited)
Coronary artery lesions occur
Exhibits hematologically strong inflammation findings in the acute stage
Blood platelets increase in the convalescent stage
Antimicrobial agents are ineffective

Such pathogens must overcome the following challenges, in order, from low to high level of difficulty: (1) detected at a high frequency in a patient; (2) symptoms can be explained; (3) coronary artery lesions (CALs) can be explained; and (4) epidemiological conditions can be explained.

### Various Theories Regarding the Etiology

**Figure 1:**
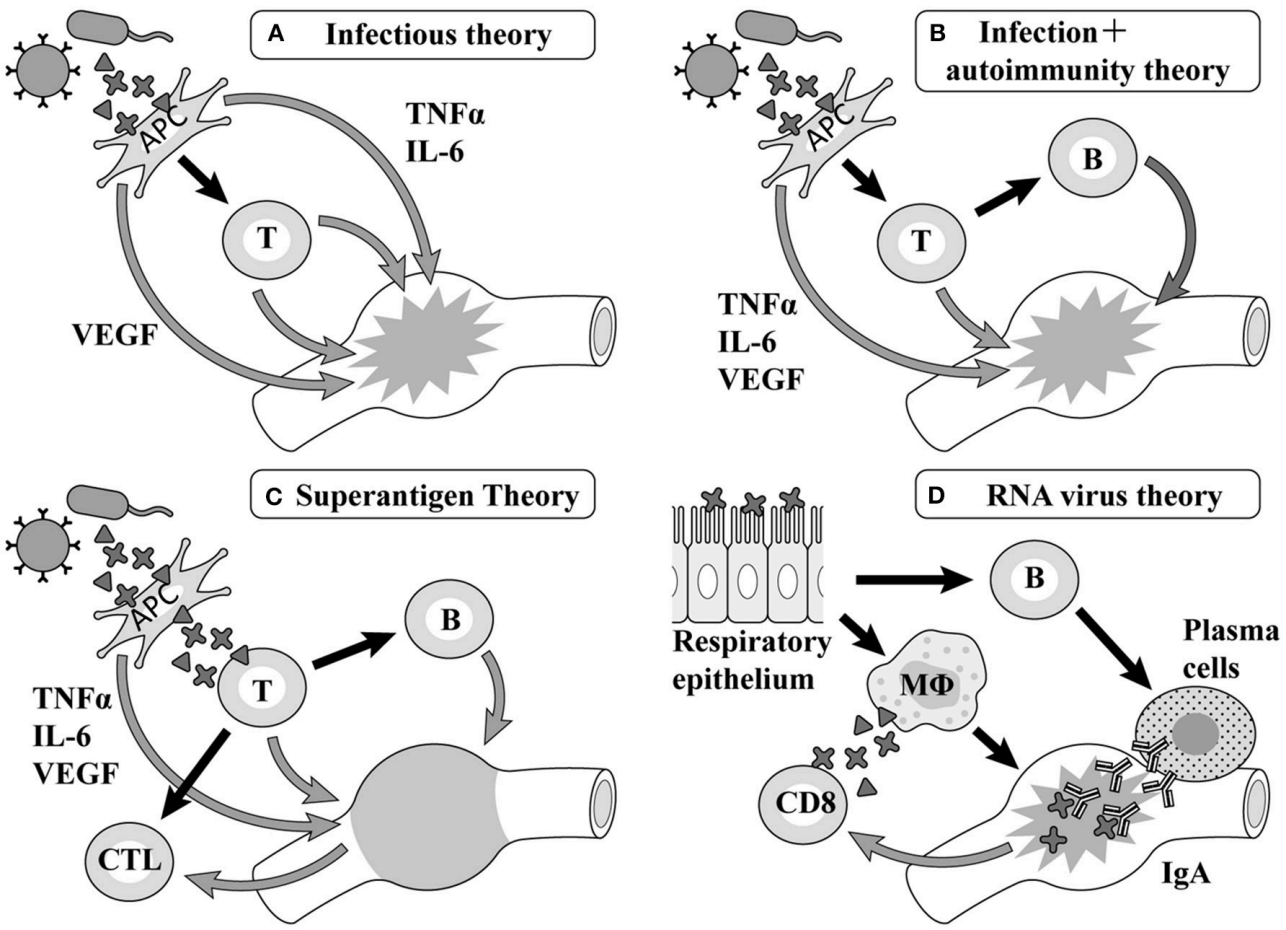
Etiology hypotheses and mechanisms. TNF, tumor necrosis factor; IL, interleukin; VEGF, vascular endothelial growth factor; APC, antigen-presenting cells; MΦ, macrophages.

A common point of view among infectious theories at an early stage is that a pathogen is recognized as an antigen-presenting cell (APC), with factors such as tumor necrosis factor (TNF) α, interleukin (IL)-6, vascular endothelial growth factor (VEGF) (9) produced by macrophage and T cells, etc. activated by macrophages causing vasculitis, leading to the formation of the pathology (Figure 1A). Although there are many pediatric diseases mainly involving vasculitis, few diseases besides KD cause CALs. Therefore, many researchers assume the existence of an autoantigen that becomes the target of these attack factors in the components of the vessel wall of small and medium-sized arteries such as the coronary artery (Figure 1B). However, the autoantigen involved remains uncertain.

#### Superantigen Theory

According to previous reports on Staphylococcus, hemolytic streptococcus, and Yersinia, the generation of superantigens is involved in the pathology of KD in each report (10–15) (Figure 1C). The primary symptoms of KD such as fever, oral cavity findings, and exanthema as well as aggravation of the serum inflammatory reaction are similar to those of diseases related to superantigens. However, the formation of CALs, as the most typical characteristic of KD, cannot be observed in diseases related to superantigens (16).

#### RNA Virus Theory

Rowley et al. have reported that the postmortem examination of KD patients revealed the invasion of many mononucleoses, CD8 positive T-cells, and IgA producing plasma cells (17), proposing a hypothesis that viruses invading mainly from the respiratory apparatus stimulate CD8 positive T-cells and B cells in the organ lymph nodes and differentiate them into IgA producing plasma cells, with these cell groups giving rise to vasculitis (Figure 1D). Recent studies have shown that respiratory viruses are detected by a PCR in up to half of KD patients (18, 19) and an ultrastructural search suggested the possibility of a respiratory virus in autopsy specimens (20, 21); however, no virus has ever been repeatedly confirmed in such studies (22).

### Superantigen + Heat-Shock Protein Theory

The authors have hypothesized the gastrointestinal (GI) tract microbiota could be involved in KD because of the following reasons: (1). GI tract is constantly exposed to a milieu of microorganisms, various antigens, and other agents, (2). It is the largest lymphoid tissue in the body, and (3). It has not been fully investigated due to some technical problems. Therefore, we focused on the mucous membrane of the GI tract with a vast area invaded by antigens and enhanced mucosal immunity, and conducted a biopsy on the mucous membrane of the duodenum of infants with KD, suggesting that certain types of antigens may have invaded that significantly activate the immunologic system of the host (23). We suspected that the possible antigens causing the disease may be a virulent alpha hemolytic streptococcus and Staphylococcus, which produce superantigens using the T-cell receptors of Vβ2 repertoire of the GI tract mucosa of infant patients 1 (24, 25). However, the problem we faced is that superantigens cannot explain the formation of coronary artery lesions and differences in race among patients with KD. In 2005, the authors conducted the following analysis (8), hinting at the theory that “superantigens cause the explosive activation of T-cells, producing autoreactive cytotoxic T-cells, which attack autoantibodies expressed on vascular endothelial cells, resulting in vasculitis” (16).

#### Knowledge Obtained From the Analysis by the Authors

**Figure 2:**
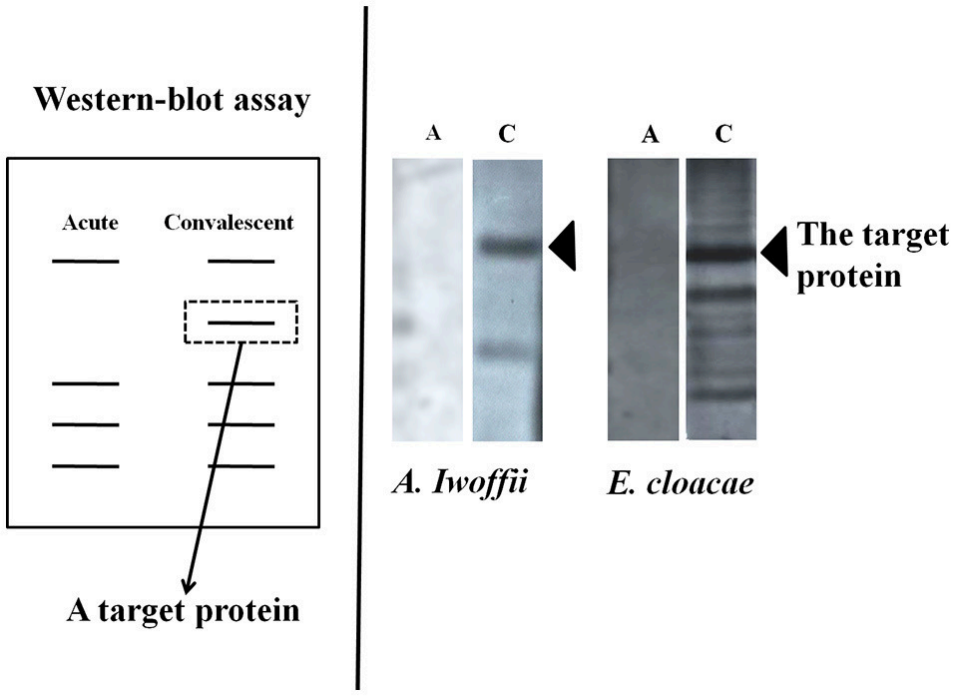
Detection of antibodies responses to bacterial products. The target proteins could be produced in the acute phase of Kawasaki disease and neutralized by intravenous immunoglobulin in the convalescent phase.

First, culture supernatants of bacteria isolated from the oral cavity or duodenal mucosa of 19 children with KD were added to peripheral blood mononuclear cells of the same host, from which we selected those promoting significant cell proliferation using ^3^H-thymidine uptake (21–23). As a result, six kinds (18 strains) of gram-positive cocci and five kinds (13 strains) of gram-negative bacteria which remarkably proliferate the peripheral blood mononuclear cells of the same patient child were isolated from the oral mucosa/duodenal mucosa of the patient. In 12 of 19 patients, both gram-positive cocci and gram-negative bacteria were isolated, including patients complicated with CALs. These bacteria showed remarkable tolerance to antibiotics. An examination of the superantigen activity by flow cytometry revealed that the isolated gram-positive cocci induced a marked increase in T cells using the Vβ2 repertoire of the host peripheral blood (26, 27). Furthermore, we detected the reactive protein between the co-culture supernatant with mononuclear cells and the host serum using the Western blotting method (28) (Figure 2). In the gram-negative bacteria, while a large amount of bacteria-specific heat-shock protein (HSP) 60 was generated in the acute phase of KD, the production of a large amount of human HSP60 was induced in the peripheral blood of the infant patients. In addition, both gram-positive cocci and gram-negative bacteria caused the secretion of inflammatory cytokine: interferon gamma (IFNγ) and TNFα, in the peripheral blood mononuclear cells of the hosts. On the other hand, gram-negative bacteria induced the production of anti-inflammatory cytokine, IL-10 in the peripheral blood mononuclear cells of the patients (29).

#### The Pathogenesis of KD Presumed From the Results of Our Analysis (Figure 3)

At first, once gram-positive cocci with superantigen activity infect the host from the mouth, they stimulate gram-negative bacteria in the upper GI tract and promote the production of HSP60. The superantigen stimulates Th1 cells causing inflammation and also induces the production of autoreactive B cells and cytotoxic T cells. With vascular endothelial cells, the stimulation of HSP60 produced by gram-negative bacteria promotes the production of human HSP 60, which plays a cytoprotective role in the nucleus and cytoplasm and is arranged so as to penetrate the membrane on the surface of vascular endothelial cells. Since part of human HSP 60 has a molecular structure derived from bacteria and the other part has molecular structure specific to human beings, human HSP 60 becomes a target of autoantibodies and cytotoxic T cells produced by autoreactive B cells generated by superantigens. The membrane of vascular endothelial cells is destroyed, causing CALs (30–34). Subsequently, human HSP60 is extracellularly released and the molecular structure derived from bacteria activates Th1 cells, while the molecular structure specific to humans activates regulatory T cells. The former further contributes to vasculitis through the secretion of IFNγ, with the latter suppressing the excessive activation of Th1 cells through the secretion of anti-inflammatory cytokine, IL-10 (35). Epidemiological and immunopathological studies have suggested that HSP60 autoantibodies can cross-react with bacterial and self-HSP 60 and induce cytotoxic damage of stressed endothelial cells, resulting in coronary atherosclerosis (31–34). These theories appear applicable to the pathogenesis of coronary lesions of KD considering the high HSP 60 expression in endothelial cells together with the serological detection of bacterial and self-HSP in patients with KD (36–38). Vascular surface-expressed HSP60 the transfer of which from the cytoplasm or mitochondria could be induced by bacterial HSP60 stimulation, can be recognized by circulating anti-HSP autoantibodies or cytotoxic T lymphocytes targeting autoantigens. The incidence of coronary lesions in patients with KD may depend upon how strongly causative agents can induce the initial immune activation that elicits autoreactive T cells and importantly, the number of self-HSP molecules they can evoke from the cytoplasm or mitochondria to the vascular surface. Gram-negative microbes appeared to trigger more self-HSP than Gram-positive cocci. Actually, Gram-negative microbes such as *N. mucosa* coexisting with Gram-positive cocci have been isolated in KD patients with vascular involvement.

**Figure 3:**
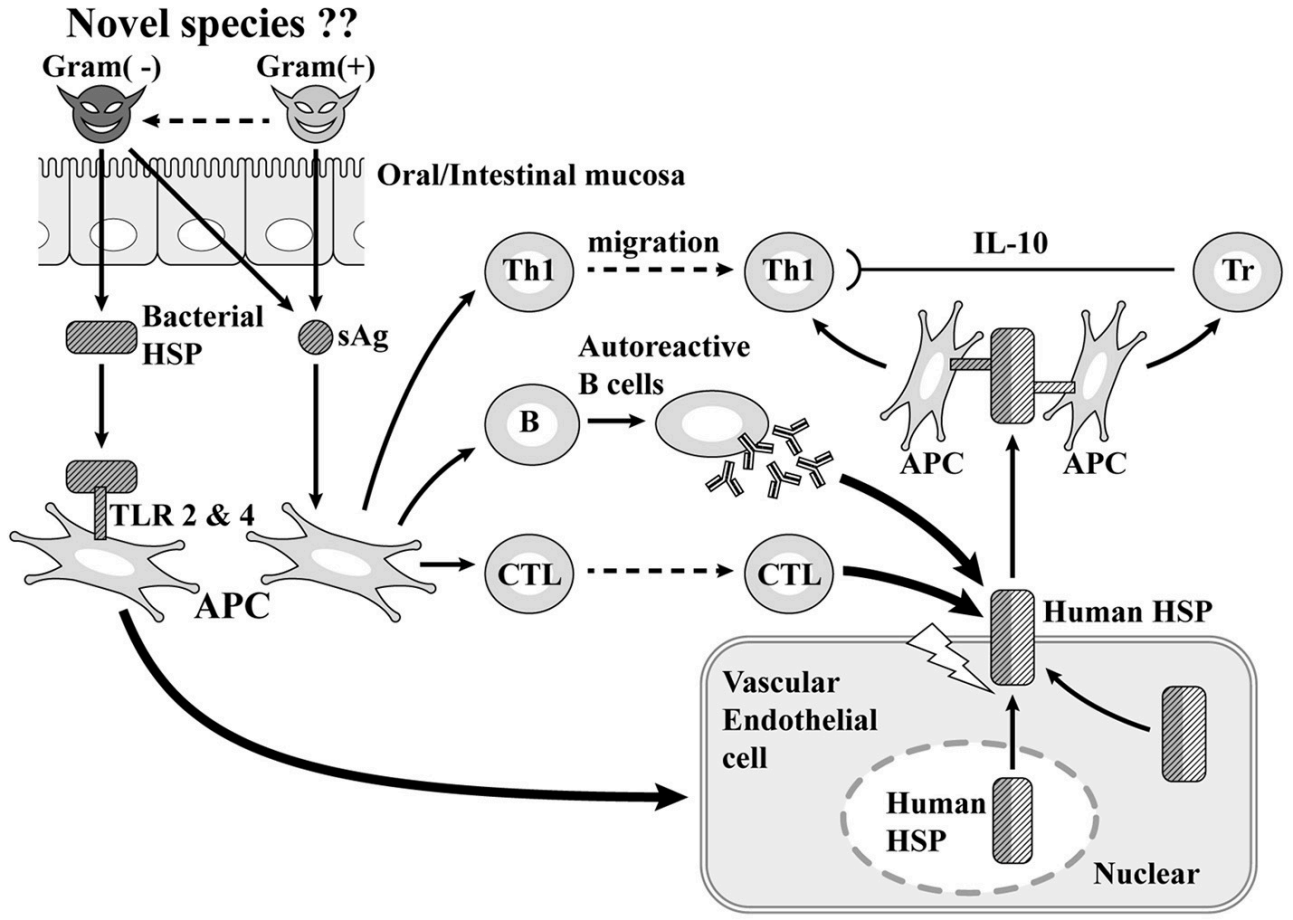
Superantigen (sAg) + coronary artery lesion due to heat-shock protein (HSP; our theory). TLR, Toll-like receptor; Tr, regulatory T cell.

### Why the Incidence Is High in Japanese People

**Figure 4:**
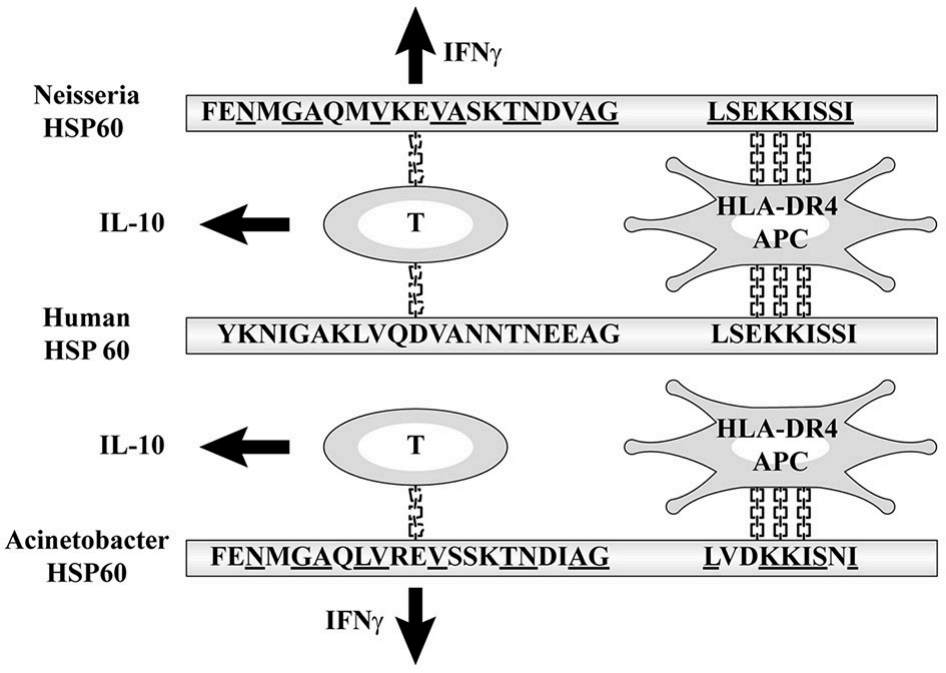
Affinity between HLA-DR4 and the binding site with MHC class II molecules on bacterial HSP60.

The amino acid sequences of *Neisseria* HSP60 221–231 and 255–269 had high homology (60 and 50%, respectively) with self-HSP 60 246–256 and 280–294, which are core protein epitopes with high capability to bind to human class II molecules, to induce the production of IFNγ and IL-10, respectively (29). These sequences were demonstrated to have high affinity with HLA-DRB1 ^*^0401, the gene products of which are recognized by HLA-DR4, which is detected by DR-peptide binding assays. Therefore, the higher incidence of KD is due to the affinity between the binding site with MHC class II molecules on bacterial HSP60 and HLA-DR4 (39–41) (Figure 4). The frequency of having this subclass of MHC class II of HLA-DR4 varies between races and has been reported as being typically high in Japanese people (42). This may account for the high incidence of KD among Japanese. Korea and Taiwan have the second and third highest annual incidences of KD in the world, which may support this hypothesis (43, 44).

Regional differences in the risk-allele frequencies of some susceptibility single nucleotide polymorphisms (SNPs) have been identified in genes such as caspase-3 (CASP3) and inositol 1,4,5-triphosphate kinase-C(ITPKC) (45, 46); however, none of the associated parameters proved to be informative in predicting the onset of KD or the development of coronary artery complications (47, 48).

### Antimicrobial Therapy to KD

The background of our study was based on the hypothesized mechanisms underlying the efficacy of intravenous immunoglobulin (IVIG), which include neutralization of the etiologic agents as well as the immunomodulation of T cell regulation and a reduction in the productions of inflammatory cytokines, such as TNFα (49). The levels of IL-6, IFNγ, and TNFα have been reported to be significantly increased before IVIG treatment. While those of IL-6, IL-10, and IFNγ rapidly decreased after treatment (50). The level of TNFα significantly reduced after treatment in KD patients without coronary artery lesions (CALs); however, it was still high in those with CALs and in patients with IVIG-resistant disease (51). Corticosteroids have been considered for such patients as a representative adjunctive therapy that has the potential to non-specifically reduce inflammatory cytokine production; however, such treatment also has the potential to induce hypercoagulopathy at the time of thrombus formation in CALs. Most the other agents used for adjunctive therapies, such as single infusion of infliximab and cyclosporine, are administered because they are effective for reducing inflammatory cytokine production; however, these are nothing more than symptomatic treatments.

In our study, we found particular Gram-negative microbes producing HSP 60 and several Gram-positive cocci possessing superantigenic properties on the surface of the GI tract that might be involved in the onset of KD. We showed that these organisms were all resistant to commonly used antibiotics, except for sulfa-methoxazole trimethoprim (SMX-TMP). We used SMX-TMP for seven cases of KD that were unresponsive to IVIG and studied the antipyretic potency of this treatment. In six out of the seven cases, antipyretic potency was observed without side effects within 2 days of the initial administration (52). Antimicrobial therapy using SMX-TMP may therefore represent a novel strategy for treating cases of KD that are unresponsive to IVIG.

### Future Prospects

The key to solving the mystery of the pathology of KD is likely to be hidden in CALs as the primary lesion. Aneurysmorrhaphy has interesting characteristics in that the intima and the adventitia are invaded before the media, along with the fact that the blood vessels distributing outside the organ are invaded, although the main characteristic is the destruction of the media forming the framework. We intend to detect possible pathogens on the upper intestinal mucosa or in the peripheral blood using a highly sensitive microbial analytical system based on reverse transcription-quantitative PCR (53). Going forward, we think it is desirable to launch a multicenter collaborative project involving the registration, preservation and search for autopsy specimens, etc.

## Conclusion

Currently, among the numerous etiologies of KD, the most credible theory is that bacterial infection triggers KD. However, we should also consider the possibility of fungi and new types of viruses, which are pathogens that have not yet been paid attention to Rowley (54). To this end, the application of new technologies is expected, such as comprehensive analyses and microarrays. In addition, we would like to emphasize that therapeutic validation should be conducted for possible pathogens, including confirming the effectiveness of antimicrobial agents, etc., in order to make the etiology theory useful in practice.

## Author Contributions

The author confirms being the sole contributor of this work and has approved it for publication.

### Conflict of Interest Statement

The author declares that the research was conducted in the absence of any commercial or financial relationships that could be construed as a potential conflict of interest. The handling editor declared a shared affiliation, though no other collaboration, with the author SN at time of review.
